# Registration and Alignment Between *in vivo* Functional and Cytoarchitectonic Maps of Mouse Visual Cortex

**DOI:** 10.21769/BioProtoc.2731

**Published:** 2018-02-20

**Authors:** Jun Zhuang, Quanxin Wang, Marc Takeno, Jack Waters

**Affiliations:** Allen Institute for Brain Science, Seattle, United States

**Keywords:** Architectonic map, Retinotopic map, Registration, Cortex flattening, Tangential section, Vasculature, Cytochrome c oxidase

## Abstract

This protocol describes a method for registration of *in vivo* cortical retinotopic map with cytochrome c oxidase (CO) labeled architectonic maps of the same mouse brain through the alignment of vascular fiducials. By recording surface blood vessel pattern and sequential alignment at each step, this method overcomes the challenge imposed by tissue distortion during perfusion, mounting, sectioning and histology procedures. This method can also be generalized to register and align other types of *in vivo* functional maps like ocular dominance map and spatial/temporal frequency tuning map with various anatomical maps of mouse cortex.

## Background

The mouse visual cortex can be segregated into functionally distinct visual areas by *in vivo* retinotopic mapping (Marshel *et al*., 2011; Garrett *et al*., 2014; Zhuang *et al*., 2017) or by neuronal track-tracing techniques aided by architectonic structures (Olavarria and Montero, 1989; Wang and Burkhalter, 2007). These different visual areas have distinct response properties and corticocortical connectivity (Andermann *et al*., 2011; Marshel *et al.*, 2011; Roth *et al*., 2012; Wang *et al*., 2011 and 2012). These results suggest that mouse visual areas form segregated visual streams processing different types of visual information (Murakami *et al*., 2017; Smith *et al*., 2017). Studying the mouse visual system in the context of visual area maps is essential to understanding the organization of visual cortex. However, although the functional maps and structure maps are broadly similar, the two maps have been shown not matching perfectly (Zhuang *et al*., 2017). For example, the primary visual cortex (V1) appears as an upward triangle in both maps, but the lateral edge of V1 in retinotopic map can be up to 300 micrometers more medial than that in anatomical map (Zhuang *et al*., 2017). Since the smallest visual areas in mouse cortex are only a few hundred micrometers wide, ignoring this mismatch will potentially bias our interpretation of visual area functions. Furthermore, both types of maps vary significantly across different individuals. Therefore, to study the functions of identified visual areas, it is important to be able to reliably generate and compare functional and anatomical maps in the same animal. However, the tissue distortion during perfusion, mounting, sectioning and histological procedure makes it difficult to directly compare functional maps recorded *in vivo* with anatomical maps recorded after histology. Here we describe a method to overcome these challenges, allowing direct comparison between these two types of maps.

## Materials and Reagents

1. Sponge (Patterson Veterinary Supply, catalog number: 07-847-3539)
2. Metal clips (Universal Small Binder Clips, Universal, catalog number: UNV10200)
3. Razor Blade (VWR, catalog number: 55411-050)
4. Spatula (Fine Science Tools, catalog number: 10090-13)
5. Gelatin subbed slides (SouthernBiotech, catalog number: SLD01-CS)
6. Cover glass (Thermo Fisher Scientific, Thermo Scientific™, catalog number: 12450S)
7. Corning 500 ml filter system, 0.45 µm (Corning, catalog number: 430770)
8. Corning 24-well plate (Corning, Falcon^®^, catalog number: 351147)
9. Corning disposable Petri dish (100×15 mm, Corning, Falcon^®^, catalog number: 351029)
10. DyLight 649-labeled tomato lectin (Vector Laboratories, catalog number: DL-1178)
11. Dry ice
12. Tissue-Tek OCT Compound (Sakura Finetek, VWR, catalog number: 4583)
13. 10x phosphate buffered saline (PBS) (Thermo Fisher Scientific, Invitrogen™, catalog number: AM9625)
14. Ethyl alcohol, 200 proof (Fisher Scientific, catalog number: 16-100-826)
  *Manufacturer: Pharmco-Aaper, catalog number: 241ACS200CSGP*.
15. DPX mountants (Electron Microscopy Science, catalog number: 13512)
16. Paraformaldehyde (Sigma-Aldrich, catalog number: 441244)
17. Sodium hydroxide solution (NaOH) (1 N, Sigma-Aldrich, catalog number: S2770)
18. Hydrochloric acid (HCl) (36.5–38%, Sigma-Aldrich, catalog number: H1758)
19. Sodium phosphate monobasic (NaH_2_PO_4_) (anhydrous, Sigma-Aldrich, catalog number: S3139)
20. Sodium phosphate dibasic (Na_2_HPO_4_) (anhydrous, Sigma-Aldrich, catalog number: 255793)
21. Sucrose (Sigma-Aldrich, catalog number: S8501)
22. 3,3’-Diaminobenzidine (DAB, 1 mg/ml, Sigma-Aldrich, catalog number: D5637)
23. Trizma HCl (Sigma-Aldrich, catalog number: T5941)
24. Trizma base (Sigma-Aldrich, catalog number: T6066)
25. Cobalt(II) chloride (CoCl_2_) (Sigma-Aldrich, catalog number: 232696)
26. Cytochrome c (Sigma-Aldrich, catalog number: C2506)
27. Catalase (10,000–40,000 U/mg, 20–50 mg/ml, Sigma-Aldrich, catalog number: C30)
28. Dimethyl sulfoxide (DMSO) (Sigma-Aldrich, catalog number: D8418)
29. Xylene (Merck, catalog number: XX0060)
30. 4% (w/v) formaldehyde (4% PFA in PBS) (see Recipes)
31. 1% (w/v) formaldehyde (1% PFA in PBS) (see Recipes)
32. 0.2 M phosphate-buffer (PB) solution with 20% (w/v) sucrose stock (pH 7.4) (see Recipes)
33. 5 mg/ml DAB stock in 0.05 M Tris-HCl buffer (pH 7.6) (see Recipes)
34. Preincubation solution, 0.05 M Tris-HCl Buffer Stock Solution (see Recipes)
35. Incubation solution (see Recipes)
36. Rinse solution, 0.1 M PB with 10% sucrose (pH 7.4) (see Recipes)

## Equipment

1. Fume hood (Labcono)
2. 4 °C fridge (Panasonic Healthcare, model: SR-L6111W-PA; VWR, catalog number: 89031-974)
3. Scale (analytical balance, A&D Weighing, model: GH-252)
4. Hot plate stirrer (VWR, catalog number: 97042-646)
5. B10P Benchtop PH meter (VWR, catalog number: 89231-662)
6. Microtome (MICROM, model: Sliding Microtome HM 400R)
7. Widefield microscope for both bright field and fluorescence imaging (ZEISS, model: Axio Imager 2)
8. Dissecting microscope (Leica Microsystems, model: Leica MZ10 F)
9. Shaker (Corning, model: LSE™ Low Speed)
10. Incubator (Quincy Lab, model: Model 10 lab oven)
11. Peristaltic pump (Harvard Apparatus, model: MA1-55-7766)

## Software

1. Fiji (Schindelin *et al*., 2012) with TrakEM2 plugin (Cardona *et al*., 2012)

## Procedure

**Figure 1:**
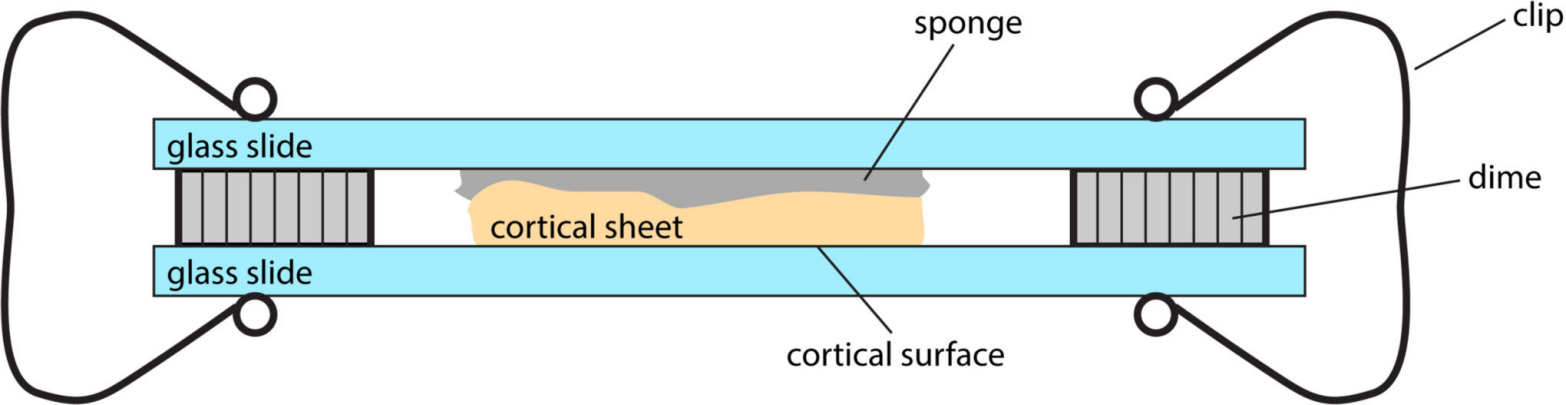
Sketch of the device used to flatten cortical sheet

**Figure 2:**
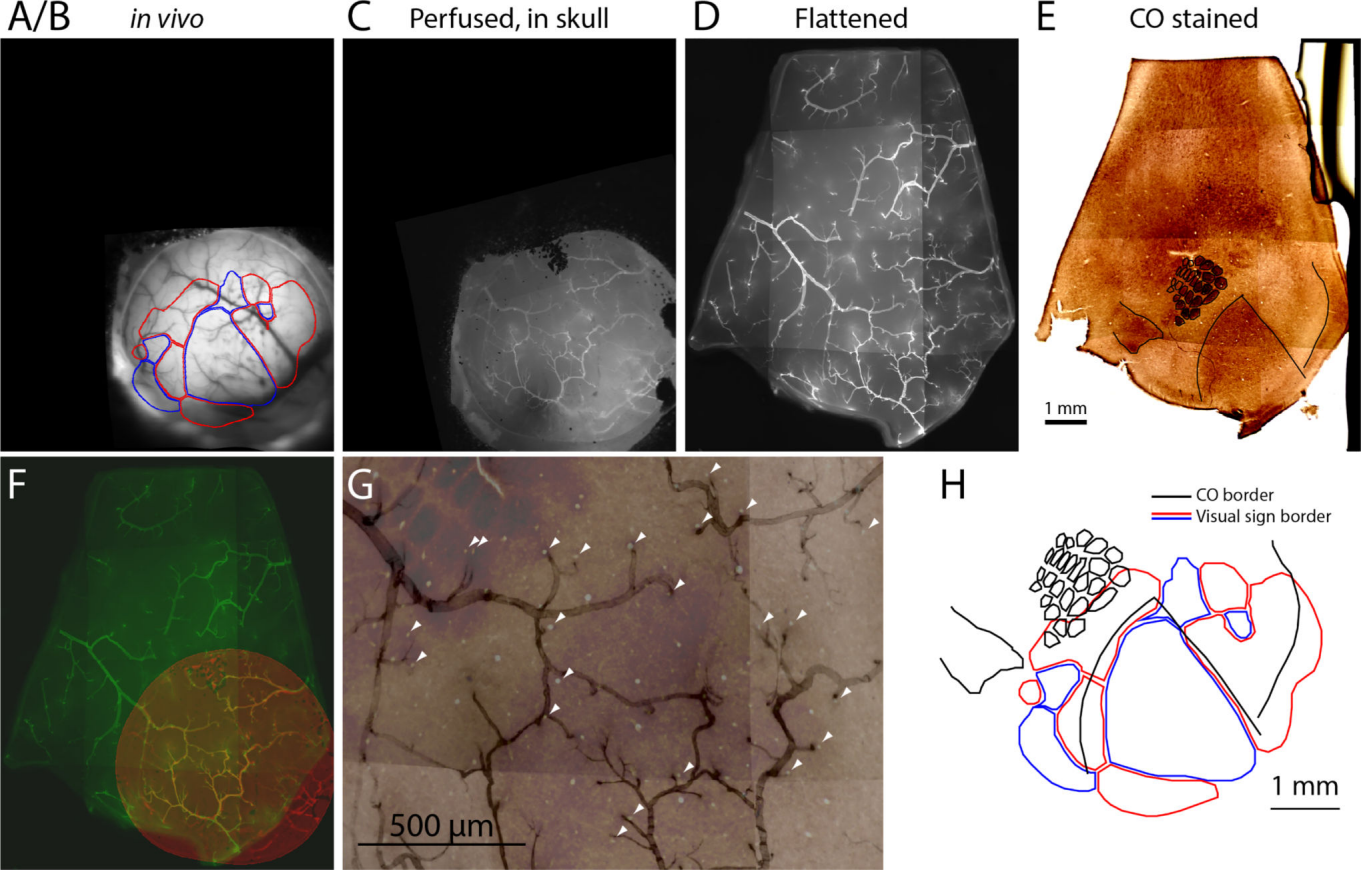
Images acquired at different steps and registration among them A–E. Images from key steps in the processing of tissue from an Emx1-Ai96 mouse, each aligned to the CO image. A/B. Brightfield image of surface vasculature with overlaid visual area map. C. Fluorescence image of whole-mount brain, after perfusion, in which a subset of the surface vasculature is labeled with DyLight 649-lectin conjugate. D. Fluorescence image of the flattened cortex. E. Bright field image of a section through layer four after CO staining. F. Overlaid fluorescence images of surface vasculature in whole-mount (red, panel C) and after flattening (green, panel D). G. Overlaid images of the surface vasculature and CO staining in posterior barrel cortex and anterior V1. The contrast of the vasculature image is inverted for clarity. Arrowheads indicate small, circular regions that do not stain for CO and likely result from transverse cuts through ascending/descending vessels. Note the alignment of these putative vessels with likely locations of ascending/descending vessels in the fluorescence image of surface vasculature. H. Field sign map (panel A/B) aligned to chemoarchitectonic borders from the CO image (panel E). Borders of primary visual cortex, auditory cortex, and of barrels in primary somatosensory cortex) were drawn manually. Modified from Zhuang *et al.*, 2017. Scale bar is 1 mm in panel H.

- *In vivo* imaging
  Make two small fiducial marks indicating the anterior and medial directions of the cranial window respectively. Record vasculature structure image of the cranial window via a fluorescence or a brightfield image (using a green wavelength may give better image contrast of blood vessels). Name it image A. Generate *in vivo* retinotopic maps through the cranial window (*i.e*., intrinsic signal Juavinett *et al*., 2017 or fluorescence retinotopic map Zhuang *et al*., 2017). Name it image B. Image A and image B should be perfectly co-registered by nature given the imaging optical axis is perpendicular to the cranial window (Figure 2A/B).
- Perfusion and cortex flattening (modified from Wang and Burkhalter, 2007)
  1. Perform mouse cardiac perfusion under isoflurane anesthesia (5% isoflurane, Gage *et al*., 2012) with the following steps of perfusion fluids.
    - Saline wash 10 ml/min for 100 ml.
    - 5 µg/ml DyLight 649 lectin 5 ml/min for 25 ml to label blood vessel.
    - Wait for 5 min for DyLight lectin to adsorb to tissue.
    - 1% PFA (see Recipes) 5 ml/min for 90 ml.
  2. With brain within the skull, acquire fluorescence image of cranial window (filter setting: 655/670 nm). The fiducial marks made in Procedure A should be visible. Name it image C (Figure 2C).
  3. Collect brain tissue. Since the animal was perfused by 1% PFA, the brain tissue will be relatively soft for cortex flattening. Be careful not to make any damage.
  4. Optional: Acquire bright field and fluorescence images (filter setting: 655/670 nm) of surface vasculature of the whole brain using a dissecting scope. The major surface blood vessels should be visible in the bright field image (can be registered with image A) and the DyLight labeled blood vessels should be visible in the fluorescence image (can be registered with image D).
  5. Isolate the cortical sheet of windowed hemisphere (the procedure can be done in a Petri dish sitting on ice). Carefully keep track of the orientation of cortical sheet. For video guidance, please see this EJN video protocol (made by Hoey Sarah, Universität Zürich): http://www.ejnnews.org/video-protocol-isolation-adult-mouse-hippocampi/.
    - Separate the two hemispheres of the brain with a razor blade. Keep the windowed hemisphere and discard the other hemisphere.
    - Cut off olfactory bulb with a razor blade.
    - Cut off brain tissue posterior to the neocortex (this include cerebellum, posterior midbrain and hind brain) with a razor blade.
    - From the medial side, gently pull out the thalamus, septum and striatum by using a spatula. Cut off these subcortical tissue.
    - Gently flip the hippocampal formation out and then separate it from cortex using a spatula.
  6. Flatten the isolated cortical sheet on a slide glass with the pia surface against the glass. Cover the other side of the cortical sheet with a piece of sponge. Cover the sponge with another piece of the slide glass. Space the two slides with two coins (we used United State dimes with thickness of 1.35 mm). Clip the both sides of slides (Figure 1).
  7. Immerse the ‘sandwich’ made in Step B6 in 1% PFA overnight (in a Petri dish in 4 °C fridge). Make sure the whole ‘sandwich’ is fully submerged.
  8. Remove 1% PFA and add 4% PFA (see Recipes) in the dish overnight.
  9. Remove 4% PFA and add 20% sucrose in the dish overnight.
  10. Remove the clips and remove the cortical sheet. Cut the outer edge of the sheet so that it is in an asymmetric shape and the orientation of the cortex (anterior, posterior, medial and lateral) is easy to identify.
  11. Take a fluorescence vasculature image (filter setting: 655/670 nm) of the flattened and cut cortex sheet before sectioning. Name it image D (Figure 2D).
- Tangential sectioning of flattened cortical sheet
  *Note: This is the crucial step and do it with extra caution*.
  1. Sufficiently cool the platform with dry ice before mounting (~10 min) the tissue and keep the platform frozen (dry ice always presented in the wells at the both end of the platform) for the whole sectioning process.
  2. Embed flattened cortex sheet in OCT with cortical surface facing up on microtome platform.
  3. Quickly put one glass slide on top of the cortex sheet before it freezes. Apply gentle pressure with fingers on the slides so that it flattens the tissue surface until it freezes.
  4. Raise and adjust the platform against the dissecting blade, until the blade is perfectly aligned with the top surface of the glass slide. Lock the platform.
  5. Lower the platform and warm the top glass slide with a finger to defrost the top surface. Remove top glass slide. Now the top surface of the cortical tissue should be perfectly parallel to the dissecting blade.
  6. Slowly raise the platform until the frozen tissue touches the blade.
  7. Check the alignment between frozen tissue surface and the blade carefully. Try very thin sections (~5 µm) to adjust alignment.
  8. Cut the first section with 150 µm thickness. This is to make sure the first section is across the whole cortical surface and contains sufficient surface vasculature for later alignment.
  9. Cut the remaining sections with 100 µm or 50 µm thickness through the whole cortex.
  10. Soak the sections in 1x PBS in sequence in a 24-well plate.
- CO staining (modified from Tootell *et al*., 1988)
  1. Wash the sections with excess PBS (pH 7.4), 3×5 min, ~40 ml per wash.
  2. Mount the sections on gelatin coated slides. Wait until completely dry.
  3. Preincubate sections with pre-incubation solution (see Recipes) at room temperature for 10 min.
  4. Rinse 4×5 min with rinse solution.
  5. Incubate sections with incubation solution (see Recipes) for 1–6 h at 37–40 °C in the dark (or foil covered).
  6. Check staining every 0.5–1 h until the reaction is sufficiently advanced and terminate the reaction by observing darkness of the tissue.
  7. Rinse sections with rinse solution (3×3 min, see Recipes).
  8. Rinse sections with dH_2_O (1×3 min).
- Dry mount (all procedure should be performed under a fume hood)
  1. Dry and defat through series of EtOH 50%, 70%, 90%, 3 min each.
  2. Wash with 100% EtOH: 2×3 min.
  3. Wash with xylene 1×5 min.
  4. Coverslip with DPX right after xylene without drying the xylene.
  5. Let the DPX solidify overnight.
  6. Take brightfield images of the sections (image series E, for example of a section across layer 4 in this series see Figure 2E, showing architectonic labeling of primary sensory cortices and retrosplenial cortex).

## Data analysis

1. Adjust the contrast and pixel resolution of images A, C, D, E so that the vasculature and cytoarchitectonic features are prominent and they all have roughly same pixel size.
2. Image B should go through same transformations as image A, so that they remain co-registered (Figure 2A/B).
3. Load all images into ImageJ TrakEM2 plugin.
4. Use *in vivo* images (image A/B) as reference and align other images progressively. Align image C to image A/B → align image D to image A/B/C → align image series E to image A/B/C/D.
  - Use non-linear transformation function (inside the TrakEM2 plugin) to align vasculature fiducials between adjacent image layers.
  - Use surface vasculature to align images A, C, D (Figure 2F).
  - Use the section outline and ascending/descending vessel cross sections to align image D and image series E (Figure 2G).
5. Once all images are co-registered, hide all the intermediate image layers and superimpose image B and the image showing the most prominent cytoarchitectonic features in image series E. The overlay image allows a direct comparison between the *in vivo* functional map and the CO labeled architectonic map (Figure 2H).

## Notes

1. The duration of Steps B2-B6 (after perfusion to flattening) should be as short as possible, longer delays may cause the brain to harden and affect the result of flattening.
2. In images C (recorded in Step B2) and D (recorded in Step B11), only a subset of the cortical surface vasculature in image A (recorded in Procedure A) will be labeled.
3. In image C recorded in Step B11, same cortical surface vasculature as that in image B should be visible.
4. When rinsing the sections during CO staining, the rotation speed of the shaker should be less than 20 rpm to avoid displacing sections from the slide.
5. DAB is GHS07, GSH08 hazardous material. Handle with caution.
6. Paraformaldehyde is GHS02, GHS05, GHS07, GHS08 hazardous material. Handle with caution.
7. The resolution of image D, image E and image series F should be high enough that the cross sections of ascending/descending vessels are visible (we used ~3 µm/pixel).
8. Sometimes inverting the contrast of some images during image alignment may help visualize the fiducials across images.
9. For image alignment, any image analysis software allowing the use of independent layers and nonlinear/warping transformations may be used; however, a suitable and widely available software is the TrakEM2 function (Cardona *et al*., 2012) in Fiji software (https://fiji.sc/, Schindelin *et al*., 2012).

## Recipes

1. 4% (w/v) formaldehyde (4% PFA in PBS, under fume hood)
  - For 1 L of 4% formaldehyde, add 800 ml of PBS to a glass beaker on a stir plate in a fume hood. Heat while stirring to approximately 60 °C. Take care that the solution does not boil
  - Add 40 g of paraformaldehyde powder to the heated PBS solution
  - The powder will not immediately dissolve into solution. Slowly raise the pH by adding 1 N NaOH dropwise from a pipette until the solution clears
  - Once the paraformaldehyde is dissolved, the solution should be cooled and filtered
  - Adjust the volume of the solution to 1 L with PBS
  - Recheck the pH, and adjust it with small amounts of dilute HCl to approximately 6.9
  - The solution can be aliquoted and frozen or stored at 2–8 °C for up to one month
2. 1% (w/v) formaldehyde (1% PFA in PBS, under fume hood)
  - For 1 L of 4% formaldehyde, add 800 ml of PBS to a glass beaker on a stir plate in a fume hood. Heat while stirring to approximately 60 °C. Take care that the solution does not boil
  - Add 10 g of paraformaldehyde powder to the heated PBS solution
  - The powder will not immediately dissolve into solution. Slowly raise the pH by adding 1 N NaOH dropwise with a pipette until the solution clears
  - Once the paraformaldehyde is dissolved, the solution should be cooled and filtered
  - Adjust the volume of the solution to 1 L with PBS
  - Recheck the pH, and adjust it with small amounts of dilute HCl to approximately 6.9
  - The solution can be aliquoted and frozen or stored at 2–8 °C for up to one month
3. 0.2 M PB solution with 20% (w/v) sucrose stock (pH 7.4, 1,000 ml)
  NaH_2_PO_4_ (anhydrous) 0.04 M: 4.8 g
  Na_2_HPO_4_ (anhydrous) 0.16 M: 22.72 g
  Sucrose: 200 g
  Adjust pH to 7.4
  Add distilled water to 1,000 ml
4. 5 mg/ml DAB stock in 0.05 M Tris-HCl buffer (pH 7.6, 100 ml, under fume hood)
  500 mg DAB
  Tris-HCl: 0.788 g
  Tris base: 0.606 g
  Adjust pH to 7.6
  Add distilled water to 100 ml
  Aliquot into 1 ml, frozen (−20 °C) for storage
5. Pre-incubation solution, 0.05 M Tris-HCl Buffer Stock Solution (500 ml) with 275 mg/L CoCl_2_ and 10% sucrose (pH 7.4, 500 ml)
  Tris-HCl: 3.305 g
  Tris base: 0.485 g
  CoCl_2_: 137.5 mg (final concentration: 275 mg/L)
  Sucrose: 50 g (final concentration: 10% w/v)
  Adjust pH to 7.4
  Add distilled water to 500 ml
6. Incubation solution (25 ml, under fume hood)
  - 0.2 M PB with 20% sucrose (pH 7.4): 20 ml
  - 4 ml DAB stock solution (5 mg/ml, final DAB concentration: 0.5 mg/ml)
  - 3 mg cytochrome c (final concentration: 0.075 mg/ml)
  - 0.008 ml catalase (final concentration: 64–640 units/ml)
  - 0.1 ml DMSO (final concentration: 0.25%)
  - Add distilled water to 25 ml (to reduce over reacting, this can be diluted to 40 ml)
7. Rinse solution, 0.1 M PB with 10% sucrose (pH 7.4) (200 ml)
  0.2 M PB with 20% sucrose (pH 7.4): 100 ml
  Add distilled water to 200 ml
